# CTP Synthase Is Required for Optic Lobe Homeostasis in *Drosophila*

**DOI:** 10.1016/j.jgg.2015.04.006

**Published:** 2015-05-20

**Authors:** Ömür Y. Tastan, Ji-Long Liu

**Affiliations:** Medical Research Council Functional Genomics Unit, Department of Physiology, Anatomy and Genetics, University of Oxford, Oxford OX1 3PT, United Kingdom

**Keywords:** CTP synthase, Cytoophidium, *Drosophila*, Central nervous system, Neuroepithelial stem cell

## Abstract

CTP synthase (CTPsyn) is a metabolic enzyme responsible for the *de novo* synthesis of the nucleotide CTP. Several recent studies have shown that CTPsyn forms filamentous subcellular structures known as cytoophidia in bacteria, yeast, fruit flies and humans. However, it remains elusive whether and how CTPsyn and cytoophidia play a role during development. Here, we show that cytoophidia are abundant in the neuroepithelial stem cells in *Drosophila* optic lobes. Optic lobes are underdeveloped in *CTPsyn* mutants as well as in *CTPsyn* RNAi. Moreover, overexpressing *CTPsyn* impairs the development of optic lobes, specifically by blocking the transition from neuroepithelium to neuroblast. Taken together, our results indicate that CTPsyn is critical for optic lobe homeostasis in *Drosophila*.

## Introduction

CTP synthase (CTPsyn) catalyses the rate-limiting stage of the *de novo* biosynthesis of CTP, one of the basic nucleotides. Nucleotides not only serve as the building blocks that make up DNA and RNA, but also play a role in energy transfer, intracellular signaling, the oxidation-reduction reaction and biosynthetic reactions. CTP synthesis starts by using glutamine and aspartate to form the base orotate in the cytoplasm, and UMP is then synthesised from orotate and phosphorylated to become UTP. CTP synthase aminates the UTP through the use of glutamine and generates CTP.

In 2010, three studies reported that in bacteria, yeast, fruit flies and rats *CTPsyn* is compartmentalised in cytoophidia (Greek for “cellular snakes”, and also known as “CTPsyn filaments” or “cytoplasmic rods and rings”) (Ingerson-Mahar et al., 2010; Liu, 2010; Noree et al., 2010). Subsequently, cytoophidia have been found in human and other mammalian cells (Carcamo et al., 2011; Chen et al., 2011), and they therefore seem to be conserved during natural selection to a high extent (reviewed by Liu, 2011). Recent work from our lab and others suggest that filamentation of CTPsyn into cytoophidia allows ultrasensitive control of enzymatic activity by compartmentalising excess enzymes in a conformationally restricted form (Aughey et al., 2014; Barry et al., 2014; Noree et al., 2014; Petrovska et al., 2014).

In *Drosophila*, our findings were that the abundance and length of cytoophidia vary in different tissues (Liu, 2010). In follicle cells, cytoophidia shorten or disappear when *CTPsyn* is knocked down (Chen et al., 2011), but, on the other hand, when *CTPsyn* is over-expressed in follicle cells, the length of cytoophidia increases dramatically (Azzam and Liu, 2013). The ectopic expression of CTPsyn-GFP in embryos may induce the formation of detectable cytoophidia in many embryonic cells (Azzam and Liu, 2013). Recent work from our laboratory has shown that a cytoophidium-forming *CTPsyn* transcript is expressed to a high degree during larval development (Azzam and Liu, 2013), though it remains unclear whether CTPsyn plays any role in brain development.

In the optic lobe region of the *Drosophila* larval brain, neuroepithelial (NE) stem cells first increase the cell population by symmetric cell division and then differentiate into neuroblasts (NBs) that undergo asymmetric division to generate medulla neurons (Hofbauer and Campos-Ortega, 1990; Egger et al., 2007). These sequential events are similar to the cell switch from NE stem cells to neuron or glial cells in the developing mammalian cerebral cortex, where NE cells proliferate through symmetric division in which one cell gives rise to identical daughter cells, followed by the neurogenesis in which a subset of cells becomes restricted to a neuronal or glial lineage (Weissman et al., 2001; Farkas and Huttner, 2008). To make the switch, NE cells begin down-regulating their epithelial features similar to NE cells in the *Drosophila* optic lobes (Götz and Huttner, 2005; Huttner and Kosodo, 2005; Kriegstein et al., 2006; Merkle and Alvarez-Buylla, 2006). Therefore, the presence of cytoophidia in NE stem cells and their disassembly upon NE to NB transition provides a great model to study CTP synthase function in a developmental context.

Here, we show that cytoophidia are abundant in the NE stem cells of *Drosophila* optic lobes, and that *CTPsyn* is required for proper optic lobe development in the central nervous system (CNS). We found that *CTPsyn* mutants have smaller larval brains, with the optic lobes as the most underdeveloped regions in the CNS. Surprisingly, overexpression of *CTPsyn* leads to optic lobe defects. Together, our data suggest that optic lobe development is sensitive to the *CTPsyn* dosage.

## Results

### Cytoophidium-forming *CTPsyn* is abundant in NE stem cells in the larval brain

**Fig. 1:**
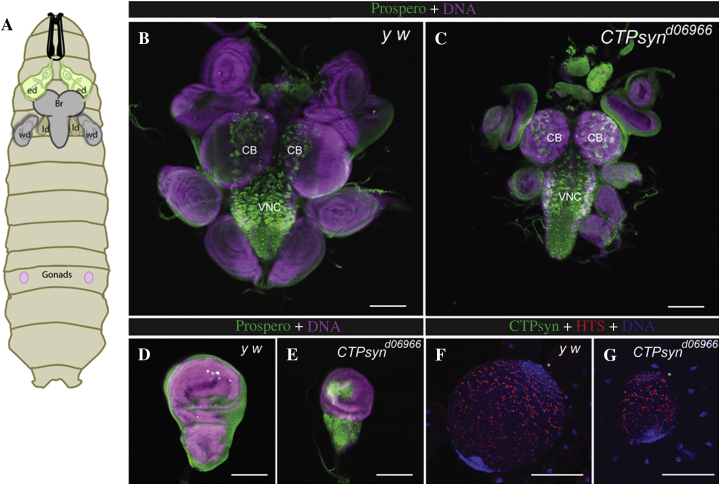
Mutations in cytoophidium-forming isoform of CTP synthase result in growth defects in larval mitotic tissues. **A:***Drosophila* larval internal organs are shown. The larval brain (Br) is located in the anterior of the larvae, with the eye (ed) and leg discs (ld) attached to it. The wing discs (wd) are also located close to the larval brain (larvae schematics modified from Jeibmann and Paulus, 2009). **B** and **C:** Wild-type and *CTPsyn*^*d06966*^ mutants were stained for Prospero, a marker for differentiated stem cells daughters, and Hoechst 33342 for nuclei. *CTPsyn*^*d06966*^ mutants have smaller larval brains with smaller optic lobes than wild-type larval brains. Most of the Prospero-positive cells in the central brain (CB) and ventral nerve cord (VNC) were, however, present in the *CTPsyn*^*d06966*^ mutants. Eye (ed) and leg discs (ld) in *CTPsyn* mutant larvae were also smaller than those in control *y w* larvae. **D** and **E:** wing discs (wg). **F** and **G:** the testes in *CTPsyn*^*d06966*^ mutant **(G)** were smaller than those in *y w* animals **(F)**. HTS, Hu-li tai shao, was used as a marker for fusome in testes, and the anterior ends of larval testes are marked with asterisks. Scale bars, 100 μm.

In order to better understand the function of *CTPsyn* during development, *CTPsyn* mutant phenotypes were characterised in *Drosophila* larval tissues. Larval tissues were stained with a neuronal differentiation marker (Prospero), and it was found that *CTPsyn* mutant larval brains had smaller optic lobes as compared to wild-type larval brains (Fig. 1A–C). In addition, we observed that imaginal discs and gonads in *CTPsyn* mutant larvae underwent a dramatic decrease in size (Fig. 1D–G).

**Fig. 2:**
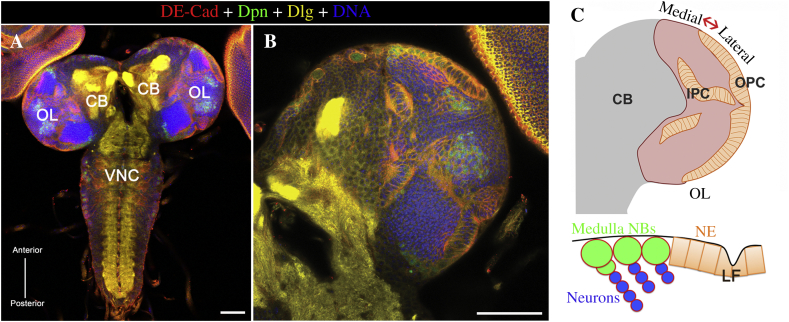
Central nervous system of *Drosophila* larva. **A:** The central nervous system (CNS) of a *Drosophila* larva. **B:***Drosophila* optic lobe stained with DE-cadherin (DE-Cad), Deadpan (Dpn), Discs large (Dlg) and Hoechst 33342. Scale bars, 50 μm. **C:** Cartoon showing NE to NB transition in a cross-sectioned optic lobe. CB, central brain; OL, optic lobe; VNC, ventral nerve cord; OPC, outer proliferation centre; IPC, inner proliferation centre; NB, neuroblast; NE, neuroepithelial; LF, lamina furrow.

**Fig. 3:**
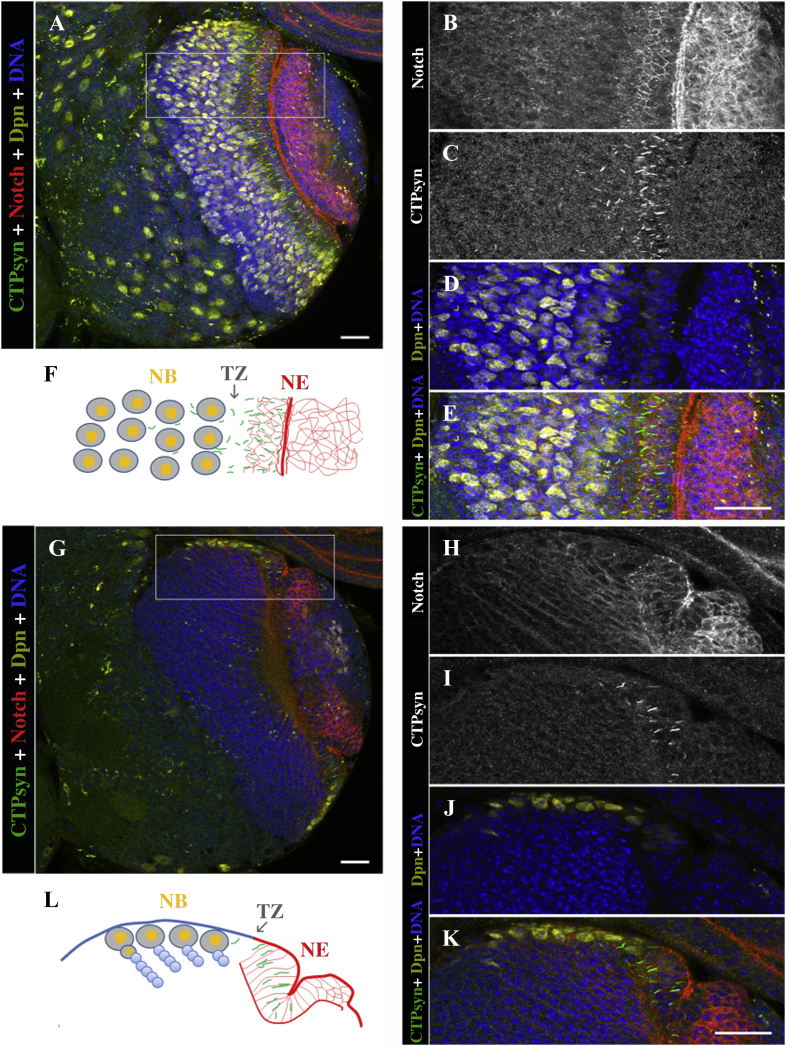
Abundance of cytoophidia in NE stem cells in the larval brain. **A–L:** The wild-type larval brains were labelled with CTPsyn (green) for cytoophidia, Notch (red) for NE, Dpn (yellow) for NBs, and Hoechst (blue) for DNA. CTPsyn is expressed throughout the larval brain and is present in the cytoophidia form in the optic lobe NE. **A–F:** Frontal view of a control optic lobe. Close-up images of the outlined areas in **(A)** are shown in **(B–E)**, showing presence of cytoophidia in the frontal view. **F:** Schematic of the outlined area in **(A)** depicting presence of cytoophidia in NE. **G–L:** Cross section of a control optic lobe showing NE to NB transition and the disappearance of cytoophidia after the TZ, depicting the enrichment of cytoophidia in the NE region (Notch, red) and its absence in the medulla NBs (Dpn, yellow). Close-up images of the outlined areas in **(G)** are shown in **(H–K)**. **L:** Schematic of the outlined area in **(G)** depicting presence of cytoophidia in NE. **K** and **L:** Cytoophidia are present in NE cells and also in the TZ, but missing in the Dpn-positive medulla NBs. **C** and **I:** Cytoophidia disappear shortly after NE to NB transition. NB, neuroblast; NE, neuroepithelia; TZ, transition zone. Scale bars, 20 μm.

In this study, we decided to focus on the larval brain (Fig. 2). We have previously shown that the cytoophidium-forming *CTPsyn* transcript is expressed to a high degree in larval tissues (Azzam and Liu, 2013). Immunostaining of the third instar larval brains (Fig. 3A–F) with antibodies against CTPsyn detected abundant cytoophidia in the NE, which also showed a very intense signal with an antibody against Notch intracellular domain. Cytoophidia disappeared in the transition zone (TZ) and in the medulla NBs, which could be labelled by Deadpan (Dpn) (Fig. 3G–L).

### *CTPsyn* mutations result in defects in optic lobes

**Fig. 4:**
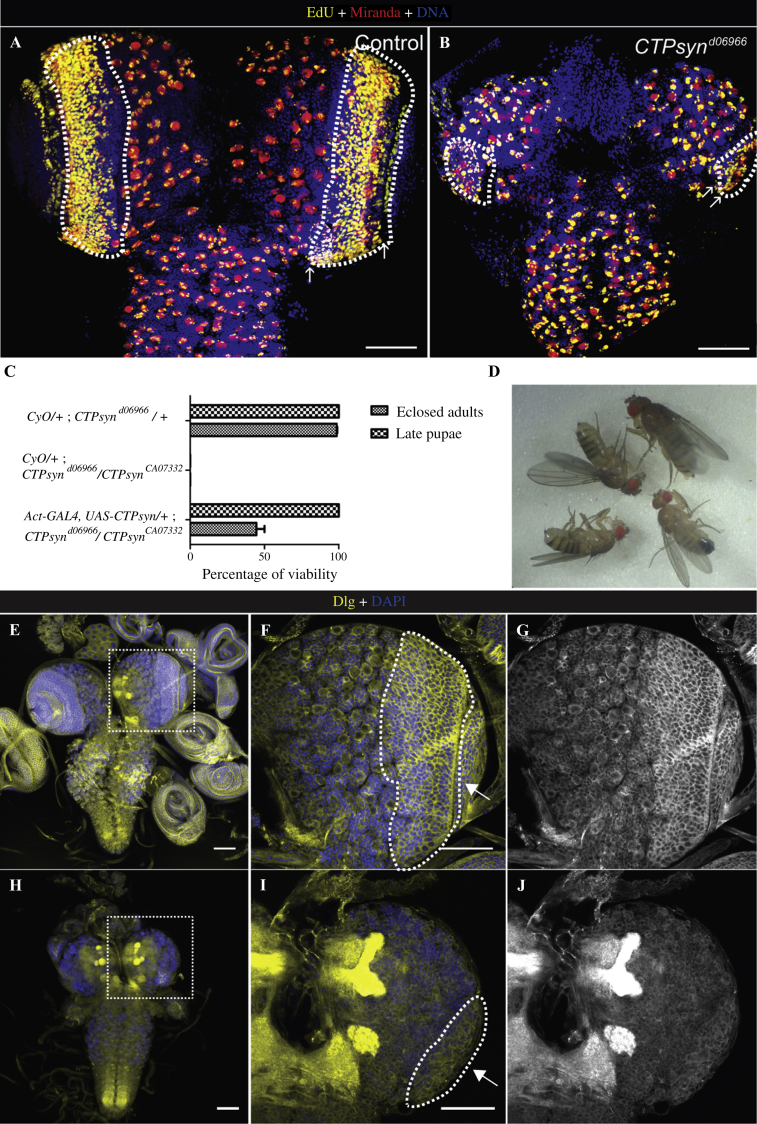
*CTPsyn* mutations impair optic lobe development. **A** and **B:** Third instar larval brains were treated with nucleoside analogue EdU (5-ethynyl-2′-deoxyuridine, yellow, a proliferation marker) and stained with Miranda (red, an NB marker) and Hoechst 33342 (blue) for DNA. **A:** Control brain lobes showing the dome-shaped medulla with abundant EdU^+^ cells. **B:***CTPsyn* mutant brain lobes are largely missing the medulla NB region, shown by the lack of an EdU^+^ region. The thickness of NE+NB region is shown by arrows. **C:** The defects in *CTPsyn* mutants are rescued by full length *CTPsyn* expressed under a ubiquitous driver, *Actin5C-GAL4* at 25°C. The *CTPsyn*^*d06966*^*/+* flies are fully viable; the transheterozygotes *CyO/+*; *CTPsyn*^*d06966*^*/CTPsyn*^*CA07332*^ are lethal at third instar larval stage. Transgenic expression of full length *CTPsyn* rescues the lethality up to late pupae formation with 50% eclosing as adults (*n* > 100). **D:** Adult flies carrying the *Actin5C-GAL4*, *UAS-CTPsyn/+*; *CTPsyn*^*d06966*^*/CTPsyn*^*CA07332*^. **E–G:***CTPsyn* brain phenotypes are rescued by full length *CTPsyn*. A *CTPsyn* mutant larva with ubiquitous expression of CTPsyn (genotype *Actin5C-GAL4*, *UAS-CTPsyn/+*; *CTPsyn*^*d06966*^*/CTPsyn*^*CA07332*^) has a normal brain showing the optic lobe (outlined area, arrow). **F** and **G:** Zoom-in images of the outlined area in **(E)**. **H–J:***CTPsyn* transheterozygote (*CTPsyn*^*d06966*^*/CTPsyn*^*CA07332*^) mutant brains are stained by Dlg and DAPI (for nuclei) showing the reduced optic lobes (outlined area, arrow). Scale bars, 50 μm.

To determine how cell proliferation is affected in different parts of the larval brain, we performed a 5-ethynyl-2′-deoxyuridine (EdU) assay to label S-phase cells; followed by anti-Miranda staining, an NB marker (Shen et al., 1997). Wild-type larval brains have established proliferation patterns with EdU-positive central brain, thoracic ganglion NBs (Miranda-positive cells) and EdU-positive dome-shaped optic lobe regions (Sousa-Nunes et al., 2011) (Fig. 4A, outlined areas, arrows). In wild-type brains, EdU staining indicated proliferative cells as previously demonstrated in NBs and some ganglion mother cells (GMCs) in central brain (CB), ventral nerve cord (VNC) and the optic lobes (OL), indicating normal proliferation rates. In contrast, brains from the *CTPsyn* mutant larvae showed a significantly reduced optic lobe. The outer proliferation center (OPC) and inner proliferation centre (IPC) were largely missing, except for a small cluster of disorganised NE and NB cells (Fig. 4B, outlined areas, arrows). The outlined areas are mainly identified based on morphology due to defects in the organisation of the NE and NB cells.

**Fig. 5:**
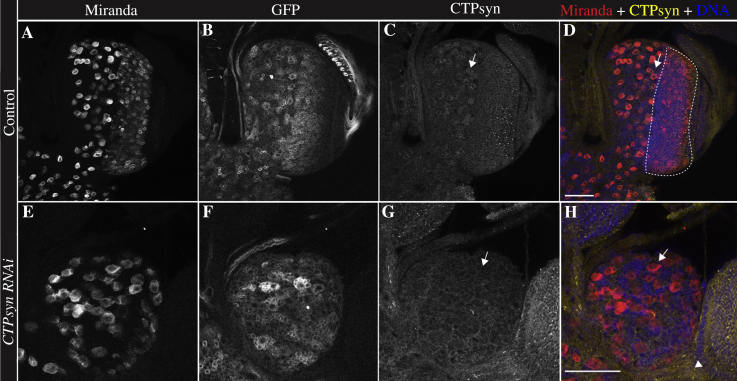
*CTPsyn* RNAi phenocopies *CTPsyn* mutants. **A–D:***Elav-Gal4/UAS-GFP* (control) flies have normal optic lobes (outlined area) and GFP shows a normal level of Elav expression throughout the optic lobe. **E–H:***Elav-Gal4/CTPsyn*^*JF02214*^ RNAi optic lobes were smaller than the control, just like the *CTPsyn*^*d06966*^ mutants. A cell with cytoplasmic CTPsyn staining is indicated by an arrow **(C** and **D)**. Cells with reduced CTPsyn staining are indicated by arrows **(G** and **H)**. Non-neuronal cells without *CTPsyn* RNAi are indicated by an arrowhead **(H)**. Scale bars, 50 μm.

There are multiple insertions in the *CTPsyn* gene region. We found that four *CTPsyn* alleles consistently showed smaller optic lobes than wild-type animals. These alleles included both P-elements (*CTPsyn^CA07332^*, *CTPsyn*^*CA06746*^) and *piggyBac* elements (*CTPsyn*^*d06966*^, *CTPsyn*^*e01207*^). The *CTPsyn* mutant phenotypes were rescued up to adulthood by a transgene ubiquitously expressing a cytoophidium-forming isoform of *CTPsyn* (Fig. 4C and D) (Azzam and Liu, 2013). Ubiquitous expression of CTPsyn in the *CTPsyn* mutant background could rescue the small brain phenotype (Fig. 4E). These results suggest that the brain phenotype is specifically caused by mutations in *CTPsyn*. Next, we were interested to see if the need for CTPsyn is tissue specific, so we knocked down *CTPsyn* in neurons using *CTPsyn*^RNAi^ with *elav-GAL4* driver, which is expressed in most neuronal and glial progenitor cells (Berger et al., 2007). RNAi mediated knockdown of *CTPsyn* also resulted in lack of optic lobes, phenocopying the *CTPsyn* mutant phenotypes (Fig. 5).

### *CTPsyn* mutations disrupt optic lobe homeostasis

**Fig. 6:**
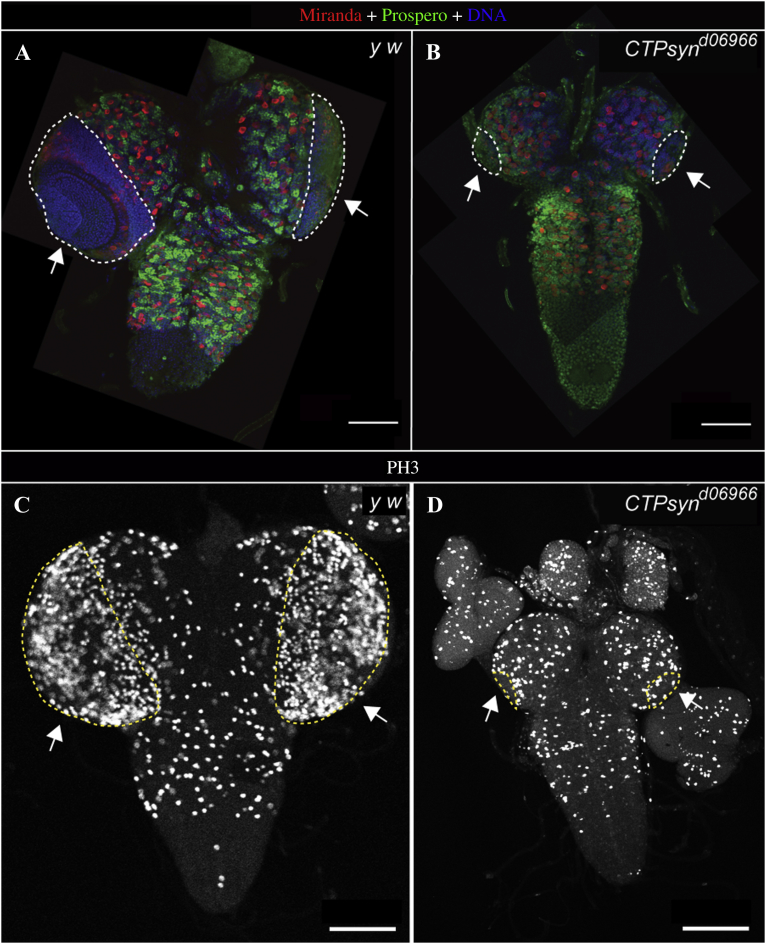
*CTPsyn* mutations result in proliferation defects in optic lopes. **A** and **B:** Third instar larval brains are stained with Miranda, an NB marker (red), and Prospero, an NB-differentiated daughter marker (green) used to visualise the optic lobes, CB and VNC NBs and their daughters. **A:** In the wild-type (*y w*), Miranda-positive NBs was observed in the CB and VNC, and their progeny was labelled with Prospero (green). **B:***CTPsyn*^*d06966*^ mutants displayed Miranda-positive NBs in the CB and VNC and Prospero-positive NB daughters. The medulla region (Hoechst dense in **(A**, outlined areas, arrows**)**) was missing in *CTPsyn* mutants **(B**, outlined areas, arrows**)**. The images were stitched up manually to show the whole brains. **C** and **D:** Third instar larval brains were stained with phosphorylated histone H3 (PH3), a mitosis marker used to visualise the highly prolific outer proliferation center (OPC) regions in both wild type and *CTPsyn*^*d06966*^ mutants. Wild-type optic lobes showed abundant PH3-stained mitotic cells around the OPC regions **(C**, outlined areas, arrows**)**, whereas these regions were not present or remarkly decreased in *CTPsyn*^*d06966*^ mutants **(D**, outlined areas, arrows**)**. Scale bars, 100 μm.

Next, we stained wild-type and *CTPsyn* mutants with Miranda and Prospero, and found that Miranda-positive NBs were present in the VNC and CB of *CTPsyn* mutants similar to wild-type, though the medulla region was missing in the *CTPsyn* mutant optic lobes (Fig. 6A and B, outlined areas, arrows).

To further detect highly proliferative OPC regions, we stained the wild-type and *CTPsyn* mutants with anti-phospho-Histone H3 (Ser10, PH3) antibody. While optic lobes in wild-type animals showed a large number of cells positive for PH3, it was observed that *CTPsyn* mutants had only scattered PH3-positive cells (Fig. 6C and D).

**Fig. 7:**
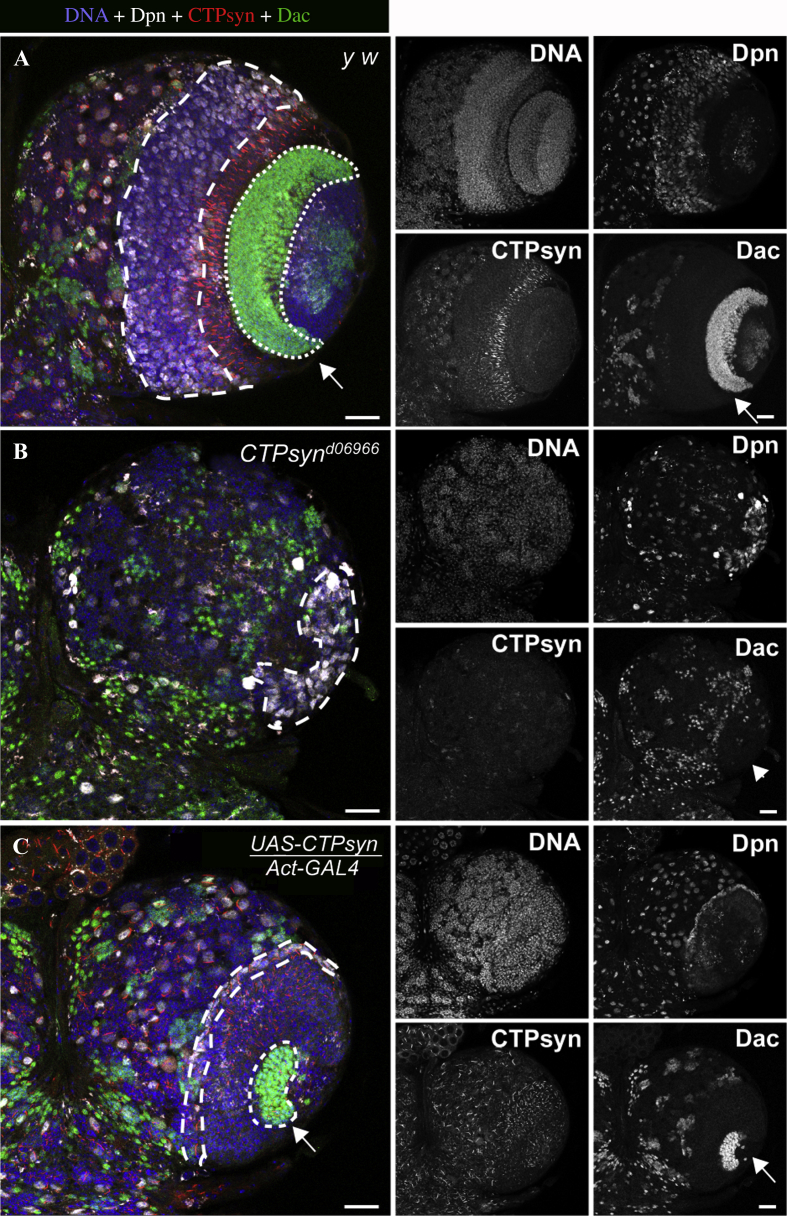
Requirement of *CTPsyn* for lamina development in the optic lobe. Third instar larval brains were stained with Dac (green) in order to visualise lamina neurons in the optic lobes, and CTPsyn (red) for cytoophidia, Dpn (white) for NBs and Hoechst (blue) for DNA. **A:** In wild-type optic lobes, Dac-positive cells form a crescent-shaped region (outlined by a dotted line, arrow) below the cytoophidium positive NE region, and the Dpn-positive medulla NB region is also present (outlined by a dashed line), evidence of a proper NE morphogenesis. **B:** No obvious lamina was detected in *CTPsyn* mutants, arrowhead showing the lack of lamina. **C:** In *CTPsyn* over-expressing optic lobes, the lamina region looked smaller (outlined by a dotted line, arrow). Scale bars, 20 μm.

The results above showed a lack of medulla region in *CTPsyn* mutant optic lobes. During optic lobe development, NEs in the OPC give rise to medulla NBs and lamina neurons. To assess the presence of a lamina, we stained control and *CTPsyn* mutant larval brains with an antibody against Dachshund (Dac). Dac, a transcriptional regulator, is required to make lamina precursor cells and lamina neurons in the *Drosophila* optic lobes (Chotard et al., 2005). In control animals, Dac-positive cells form a layer under the NE+NB region, which are the lamina precursor cells (Fig. 7A, dotted outline, arrow). Whereas in *CTPsyn* mutants, we did not observe any Dac-positive cells underneath the Dpn-positive cell cluster (NE+NB region, dashed outline) suggesting a complete disruption of lamina development (Fig. 7A and B).

In *CTPsyn* mutants, the optic lobes might be impaired from early embryonic stages. High levels of EdU incorporation in the underdeveloped regions suggest that these cells are able to enter mitosis, but that later stages of mitosis might be affected. It is also possible that the NE to NB cell fate switch is impaired, and cells become stuck in a mixed cell environment without differentiating. Further investigation is required to assess how *CTPsyn* functions in different cell populations in the larval brain.

### Over-expression of CTP*syn* impairs brain development

**Fig. 8:**
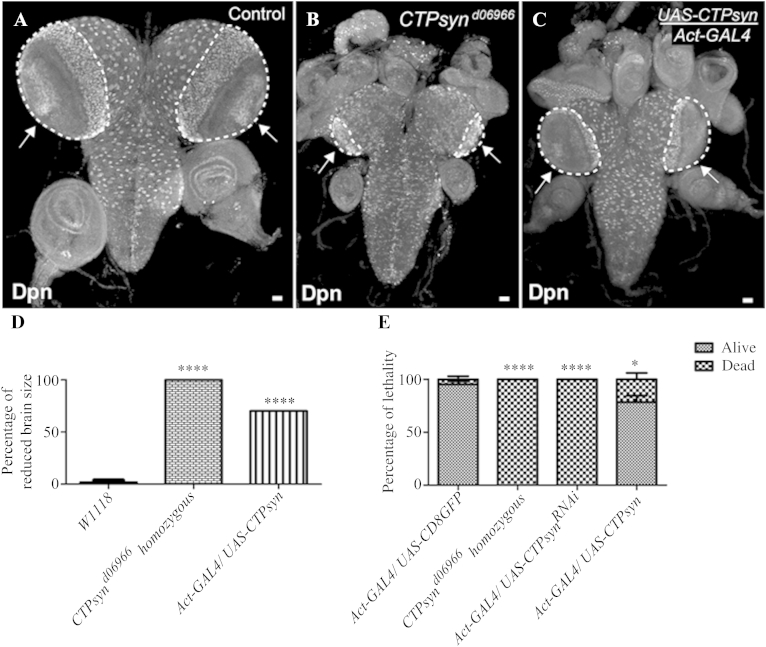
Over-expression of *CTPsyn* leads to developmental defects in the larval brain. Larval brains were stained with Dpn (white) for NBs to show the general morphology of the larval central nervous system (CNS). **A** and **B:***CTPsyn* mutant larval brains lack general organisation and have smaller optic lobes with malformed NE, and medulla regions characterised by clumped Dpn-positive cells (arrows) compared to control larval brains (outlined areas). **C:***CTPsyn* over-expression with *Actin5C-GAL4*, a ubiquitous driver leads to defects in NE differentiation. Outlined areas show the underdeveloped optic lobes with a thin layer of medulla neurons (Dpn-positive cells, arrows) in *CTPsyn* over-expressing larvae. **D:** The percentage of reduced brain size among control (*w1118*), *CTPsyn* mutant and the *CTPsyn* over-expressing larvae. ****P < 0.0001, *n* > 50. **E:** The lethality rates among control, *CTPsyn* mutant, *Act5C-Gal4>CTPsyn^RNAi^* and the *CTPsyn* over-expressing larvae. ****P < 0.0001, *P < 0.05, *n* > 100. Scale bars, 20 μm.

**Fig. 9:**
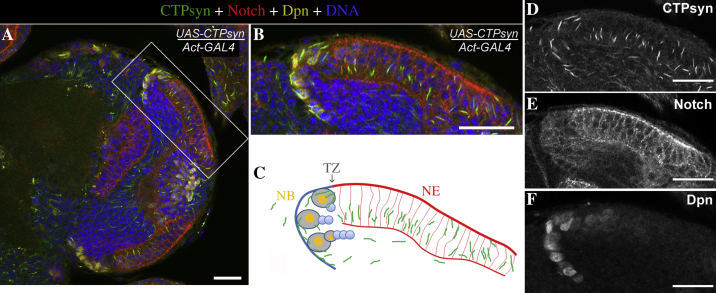
Cytoophidia persist in medulla NBs upon *CTPsyn* over-expression. **A** and **B:** The over-expression of *CTPsyn* in optic lobes results in persistent cytoophidia in medulla NBs, even after the transition zone. **C:** The schematic shows the persistence of cytoophidia (CTPsyn, green) in NE stem cells (Notch, red), transition zone cells and medulla NBs (Dpn, yellow). Scale bars, 20 μm. **D**–**F:** Individual grey channel images of (**B**) are shown for clarity. Scale bars, 20 μm.

To better understand the role of *CTPsyn* during NE morphogenesis, a transgene was over-expressed with a cytoophidium-forming isoform of *CTPsyn*. The overall body size of third instar larvae was comparable in both the *CTPsyn* over-expression and the wild-type. A closer look at the larval brains revealed that the NE and medulla NB regions did not form properly in 65%–70% of the larvae examined (*n* = 47). In *CTPsyn*-overexpressing larvae, the NE was less organised and the medulla region decreased dramatically in volume as compared to that of the wild-type (Fig. 8A and C). All *CTPsyn* mutants, with 100% lethality at third instar larval stage, had smaller brains than the controls (Fig. 8D and E). Ubiquitously over-expressing CTPsyn led to ∼30% lethality at pupae stage (Fig. 8E). Staining larval brains with antibodies against CTPsyn detected long and abundant cytoophidia not only in the optic lobes, but also in NBs in the CB and VNC, confirming that CTPsyn is indeed over-expressed in those transgenic animals (Fig. 9).

**Fig. 10:**
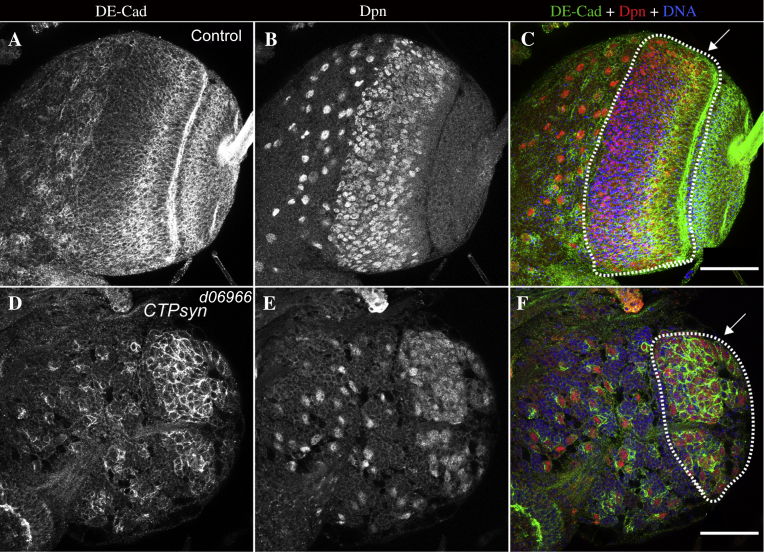
Optic lobe development is disrupted in *CTPsyn* mutants. Third instar larval brains were stained with DE-Cad (green), Dpn (red), and Hoechst 33342 (blue) for DNA. **A**–**C:** Control optic lobes with a layer of NE, bright DE-Cad staining **(**arrow, **C)** and medulla region (Dpn-positive, outlined area). **D**–**F:***CTPsyn* mutant optic lobes have a smaller medulla region (arrow and outlined area) than controls. Scale bars, 50 μm.

NE stem cells in the OPC are columnar in shape and easy to identify due to their distinct morphology (Egger et al., 2007). In addition, NE cells express adherens junction protein DE-cadherin (Dumstrei et al., 2003), whereas medulla NBs are rounded and express the marker protein Deadpan (Dpn) (Zacharioudaki et al., 2012). To examine potential abnormalities in NE cells in *CTPsyn* mutant brains, we used two antibodies against DE-cadherin (a marker for NE cells) and Notch (C17.9C6, targeting the intracellular region of Notch). We used the Dpn antibody to label medulla region NBs. DE-cadherin expression was shown to be down-regulated upon epithelial to mesenchymal transition, including the mesoderm, NBs and their neurons, and may therefore be used as a marker for NE (Tepass et al., 1996; Uemura et al., 1996). DE-cadherin is expressed in wild-type NE cells, and its expression is down-regulated as the cells differentiate further (Fig. 10A–C). In *CTPsyn* mutants, the cells that were stained with DE-cadherin showed no organisation and remained as a clump of cells (Fig. 10D–F).

**Fig. 11:**
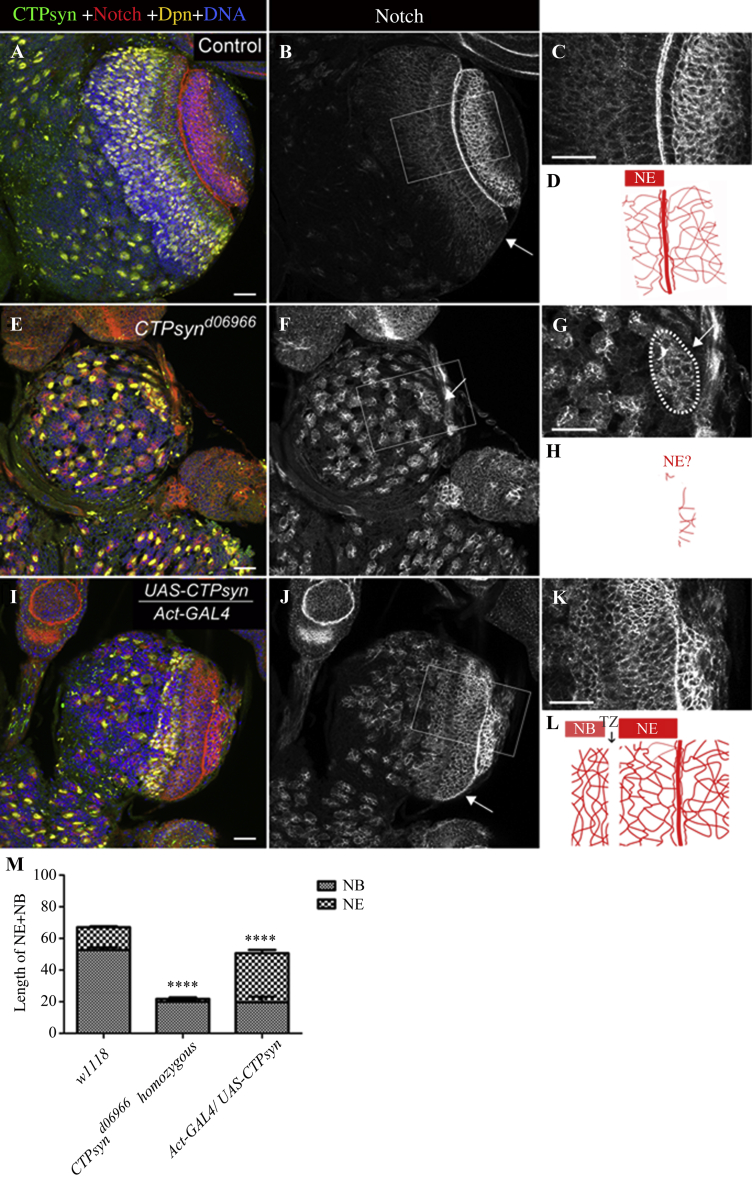
NE differentiation and the effect of changes in *CTPsyn* levels. Wild-type, *CTPsyn*^*d06966*^ and *CTPsyn* over-expressing larval brains were stained with CTPsyn (green) antibody to label cytoophidia, with Notch (red) to label NE and Dpn (yellow) antibody to label NBs in the optic lobes. **A**–**D:** In control optic lobes, cytoophidium is present in the NE cells, and the border of the NEs is enriched for Notch. **B**, **F** and **J:** NEs are to be found just below the lamina furrow, and have defined columnar shapes (arrows in **B**, **F** and **J**). **E**–**H:** In *CTPsyn*^*d06966*^ mutants, the NE and NB regions looked rudimentary and defective (outlined area in **G**). All cells in this region show Dpn staining and high levels of Notch staining. **I**–**L:** The over-expression of *CTPsyn* causes an expansion in the NE stem cell population and defects in downstream daughter differentiation, and *Actin-Gal4*/*UAS*-*CTPsyn***(I)** larval brains display underdeveloped optic lobes. High levels of Notch staining were detected in the NE and NB regions, with a temporary decline along the NE-NB boundary **(K** and **L)**. **M:** Quantification of NE to NB ratio among control, *CTPsyn* mutants and the *CTPsyn* over-expressing optic lobes (Y axis, length of NE+NB regions, *n* > 50, ****P < 0.0001). Scale bars, 20 μm.

In *CTPsyn* over-expressing larvae, the overall morphology of the larval brain was present with less medulla NBs, compared to that of the control (Fig. 8A and C). Here, we used Notch staining to follow the course of NE stem cell expansion and maintenance, and Dpn to label the medulla NBs (Fig. 11). High levels of Notch staining persisted in the expanded NE region and slightly decreased as the cells differentiated into medulla NBs and started expressing Dpn similar to controls. We quantified the NE and the NB region sizes in control, *CTPsyn* mutants and the *CTPsyn* over-expressing larvae by measuring NE and NB length at three different locations for each genotype (*n* > 50). This analysis revealed that NE was expanded in *CTPsyn* over-expressing optic lobes whereas the area of the NB region was decreased compared to control animals (Fig. 11I–L and M). The overall length of NE+NB region in *CTPsyn* over-expressing optic lobe was also less than controls (Fig. 11M). Taken together, these data pointed toward potential defects in the maintenance or expansion of the NE stem cells in the OPC, when *CTPsyn* is misexpressed.

### *CTPsyn* mutations lead to defects in glial cells

**Fig. 12:**
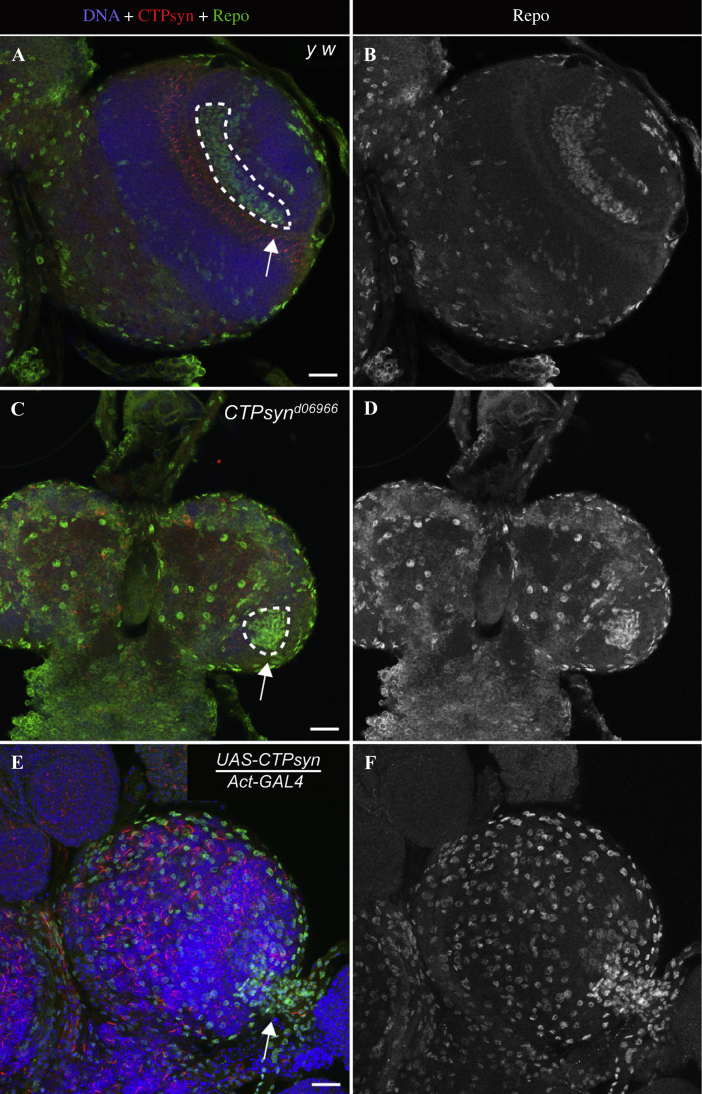
Glial cell population defects in *CTPsyn* mutants. Third instar larval brains were stained with Repo (green) to visualise the glial cell population in the optic lobes, *CTPsyn* (red) for cytoophidium and Hoechst (blue) for DNA. **A** and **B:** In control optic lobes, glial cells are present in the central brain and below the NE (outlined area, arrow). **C** and **D:** Glial cells seem to be present in *CTPsyn*^*d06966*^ mutants, though the general morphology of the glial region below the NE looks deformed (outlined area, arrow). **E** and **F:** The crescent-shaped Repo positive region is completely missing in *CTPsyn* over-expressing optic lobes, and instead Repo-positive cells may be observed at the optic stalk (arrow). Scale bars, 20 μm.

We next investigated whether glial cell populations that surround the NE cells are affected in *CTPsyn* mutants. Recent work established the presence of a glial niche for the NE cells in the *Drosophila* optic lobes (Morante et al., 2013). NE cells need to receive glial derived cues for NE cell proliferation and the NB transition (Morante et al., 2013). A subpopulation of glial cells within the optic lobes ensheath the NE, suggesting that glial cells communicate with the NE (Morante et al., 2013). Given the NE maintenance and NB differentiation defects, we asked whether glial cell populations are healthy in *CTPsyn* mutants. To answer this, we looked at Repo, a glial cell marker, in *CTPsyn* mutants compared to the control. In control flies, we observed a well-defined Repo-positive glial cell population underlying the NE stem cells (Fig. 12A and B, outlined area, arrow). Glial cells in *CTPsyn* mutant larval brains looked highly disorganised, although we could identify a cluster of Repo-positive cells in the optic lobe region (Fig 12C and D). The organisation of glial cells was also disturbed in the *CTPsyn* over-expressing larval brains (Fig. 12E and F). *CTPsyn* over-expression also resulted in shrunken lamina, as revealed by Dac staining (Fig. 7C).

## Discussion

In this study, we have observed that CTPsyn forms cytoophidia in the NE of optic lopes. Our work has shown that multiple *CTPsyn* mutants and RNAi exhibit defects in NE morphogenesis, and also that the over-expression of *CTPsyn* leads to defects in the optic lobes. These results suggest that the development of optic lobes requires the right *CTPsyn* dosage.

The biosynthesis of nucleotides is tightly regulated due to the requirement for nucleotides in DNA replication and various metabolic processes. Abnormalities in purine or pyrimidine metabolism are associated clinically with several diseases, including various degrees of mental retardation and other types of neurological dysfunction (Vanna Micheli et al., 2011). The pathogenesis of such disorders is generally explained by unspecified cellular damage or mitochondrial dysfunction. Whether there is a connection between alterations in specific enzymes such as CTPsyn and brain damage remains unclear.

The development of NE in *Drosophila* optic lobes correlates to that of the vertebrate cerebral cortex. *Drosophila* NE has been used as a model system to study primary recessive microcephaly, a neurodevelopmental disorder characterized by brain size reduction at birth accompanied by mild mental retardation. Recent work from Basto and colleagues has shown that the abnormal spindle protein (Asp), the *Drosophila* orthologue of abnormal spindle-like microcephaly associated protein (ASPM), regulates NE morphogenesis (Rujano et al., 2013). Surviving flies had smaller heads and larvae had smaller larval brains with underdeveloped optic lobes. Brain size reduction in *asp* mutants is caused by defects in spindle positioning, chromosome segregation and consequent apoptosis. In this study, we have observed that multiple *CTPsyn* mutants exhibit defects in NE morphogenesis, resembling the phenotypes of microcephaly mutants. It would be interesting to see whether defects in *CTPsyn* and the cytoophidia contribute to microcephaly.

Several recent studies suggest that the filamentation of enzymes, including CTPsyn, down-regulates their enzymatic activity (Barry et al., 2014; Noree et al., 2014; Petrovska et al., 2014). Our work on *Drosophila* and human cells support the view that inactive CTPsyn is incorporated into cytoophidia (Aughey et al., 2014), and that the polymerisation of CTPsyn into filamentous cytoophidia might stabilise the enzyme in a particular state. The enzymatic activity of CTPsyn may vary among dimers, tetramers and polymers without a dramatic change in the overall abundance of CTPsyn in the cell. The formation of cytoophidia in fast-growing cells such as NE might provide a quick response for the regulation of the enzymatic activity of CTPsyn.

*CTPsyn* over-expression also caused smaller brains compared to control. Unlike *CTPsyn* mutants, a distinct NE cell population was present in the *CTPsyn* over-expressing brains. However, the NE region was expanded compared to controls, which indicated a block in NE differentiation. It is possible that forcing CTPsyn to assemble into cytoophidia *via* over-expressing in NBs, a cell type that probably demands high enzymatic activity of CTPsyn, impairs downstream differentiation. It is conceivable that cytoophidia recruit and sequester not only CTPsyn, but also some additional proteins which might play key roles in NE differentiation.

In summary, our results suggest that the right levels of CTPsyn are crucial for *Drosophila* optic lobe development. Our study provides a potential link between the tight regulation of CTPsyn filamentation and proper organisation of the optic lobes, crucial for the brain development.

## Materials and methods

### Fly stocks

All stocks were raised at 25°C on standard cornmeal, and *y w* flies were used as control in all our experiments unless stated otherwise. The following *CTPsyn* mutant stocks were used in this study: 1) *CTPsyn*^*d06966*^ (received from the Harvard Exelixis collection), 2) *CTPsyn* RNAi line (*y1 v1*; *P{TRiP.JF02214}attP2)*, 3) *ElavGal4,UAS-GFP (P{w[+mW.hs]=GawB}elav[C155]*, P{w[+mC]=UAS-CD8::GFP.L},w[*]), 4) *Actin5C-GAL4/CyO*, *twiG4-2xEGFP* stocks (obtained from the Bloomington stock centre (Thibault et al., 2004), 5) *CTPsyn*^*CA07332*^ (P{PTT-GA}CTPsyn^CA07332^) (received from the Carnegie Protein Trap Library (Buszczak et al., 2007), and 6) UAS-*CTPsyn* (Flybase ID: FBtr0344431) rescuing transgene (used in overexpression experiments (Azzam and Liu, 2013)). The rescue crosses were set up with *Actin5C-GAL4*, *UAS-CTPsyn/CyO*; *CTPsyn*^*d06966*^*/TM6B* crossed to *+/+*; *CTPsyn*^*CA07332*^*/TM6B*. Non-Tubby, non-CyO and white^+^ (dark red eyes) flies which correspond to the genotype: *Actin5C-GAL4*, *UAS-CTPsyn/+*; *CTPsyn*^*d06966*^*/CTPsyn*^*CA07332*^, were viable.

### Lethality assays

Wild-type, *CTPsyn*^*d06966*^ and *Actin5C-GAL4*, *UAS-CTPsyn/CyO* flies were cultured at room temperature. Three biological replicates from each genotype were collected over 24 h. A total of ∼100 first instar larvae (48 h after egg collection) from each genotype were transferred to food plates with wet yeast and kept at 29°C until pupation. The number of pupae scored and plates are kept at 29°C until eclosion. The number of flies that eclosed and the number of dead pupae were scored. The percentage values were calculated and plotted on the graph.

### Immunohistochemistry

Dissected fly tissues were fixed in 4% paraformaldehyde (PFA) in 1× PBS for 10 min at room temperature (RT). The fixative was then removed, and samples were rinsed by 1× PBS and incubated in wash solution (1× PBS + 0.5% horse serum + 0.3% Triton X-100). The samples were incubated in primary antibodies for at least 12 h at RT. They were then briefly rinsed with wash solution and incubated in the DNA dye Hoechst 33342 and secondary antibodies for at least 12 h at RT. Samples were then mounted onto slides for observation under confocal microscopy.

Primary antibodies used in this study included: mouse monoclonal anti-Notch antibody (intracellular domain) C17.9C6 (1:100), mouse monoclonal anti-Repo 8D12 (1:100), mouse monoclonal Abdac1-1 antibody for Dachshund (1:100) from Developmental Studies Hybridoma Bank (DSHB, USA), rabbit anti-CTP synthase y88 (sc-134457, 1:1000 Santa Cruz BioTech, USA); rabbit anti-Prospero (1:1000, a gift from Denan Wang), guinea pig anti-Dpn (1:10000, a gift from James B. Skeath), rabbit anti-DE-cadherin (sc-33743, 1:1000 Santa Cruz BioTech, USA). Secondary antibodies used in this study were anti-mouse, rabbit, guinea pig or goat antibodies labelled with Alexa Fluor^®^ 488, Cy3 or Cy5 (1:1000, Jackson Immuno Research Laboratories, USA).

### EdU labelling of *Drosophila* larval tissue

The EdU staining was performed using the Click-iT^®^ Plus EdU Alexa Fluor^®^ 647 Imaging Kit following manufacturer's protocol (Cat. No. C10640, Life Technologies, UK) apart from following changes. Third instar wandering larvae were dissected in Grace's Insect Medium (Cat. no. 11605-045, Life Technologies), and the anterior portion was inverted to expose the imaginal discs and the larval brain. The medium was replaced with fresh medium containing EdU (5 ug/mL final concentration) from the Click-iT^®^ Plus EdU Alexa Fluor^®^ 647 Imaging Kit (Cat. No. C10640, Life Technologies) and incubated for one hour at RT. After incubation, media containing EdU was removed, 4% PFA was added and the brains were fixed for 15 min at RT. These were then washed twice with 1 mL 3% BSA in 1× PBS. After washing, 1 mL of 0.5% Triton X-100 in PBS was added and the brains were then incubated for 20 min at RT. Reaction cocktails, including 1× Click-iT^®^ reaction buffer (430 μL), CuSO_4_ (Component H) (20 μL), Alexa Fluor^®^ azide (1.2 μL) and reaction buffer additive (50 μL), were prepared. The 0.5% Triton X-100 in 1× PBS was removed and the brains were washed twice with 1 mL 3% BSA in 1× PBS. 250 μL Click-iT reaction cocktail was then added to each sample on a rocker and incubated for 30 min, protected from light, at which point the cocktail was removed and the brains washed once with 1 mL 3% BSA in PBS. For antibody or DNA staining, the procedures described above were followed.

### Microscopy

Images were acquired under 20× and 63× objectives using a laser-scanning confocal microscope (Leica TCS SP5II, Leica Microsystems, UK).

### Statistical analysis

Raw data were entered into Prism (v5, GraphPad, CA) and used to produce graphs. The error bars represent the standard error of the mean values. To test the significance, two-way ANOVA tests were performed followed by Bonferroni post tests to check for significant differences between different groups. Significant differences were attributed for *P* < 0.05.
